# Supplementary material for: Capsiate Intake with Exercise Training Additively Reduces Fat Deposition in Mice on a High-Fat Diet, but Not without Exercise Training
Source: Int J Mol Sci. 2021 Jan 14;22(2):769. doi: 10.3390/ijms22020769 (PMC7828664; doi:10.3390/ijms22020769)
Supplement: Supplementary file 1 [file ijms-22-00769-s001.zip › Supplementary material S1~S4/S1,S2.pdf]

Class	Ingredient	Mass /g
Protein	Casein, lactic, 30 mesh	200
Protein	Cystine, L	3
Carbohydrate	Lodex 10	125
Carbohydrate	Sucrose, fine granulated	72.8
Fiber	Sloka Floc, FCC200	50
Fat	Lard	245
Fat	Soybean Oil, USP	25
Mineral	S10026B	50
Vitamin	Choline bitartrate	2
Vitamin	V10001C	1
Dye	Dye, blue FD&C #1, Alum. Lake 35 – 42 %	0.05

Table S1. Formulation of the high-fat diet

Protein	20 % of total kcal
Fat	60 % of total kcal
Carbohydrate	20 % of total kcal
Energy density	5.21 kcal/g

Table S2. Calorific value of the high-fat diet
