# Supplementary material for: Capsiate Intake with Exercise Training Additively Reduces Fat Deposition in Mice on a High-Fat Diet, but Not without Exercise Training
Source: Int J Mol Sci. 2021 Jan 14;22(2):769. doi: 10.3390/ijms22020769 (PMC7828664; doi:10.3390/ijms22020769)
Supplement: Supplementary file 1 [file ijms-22-00769-s001.zip › Supplementary material S1~S4/S3.pdf]

Weekend	1st	2nd	3rd	4th	5th	6th	7th	8th
Duration (min)	20	30		40		50		60
Velocity (m/min)	13		14		15		16	
Slope (°)	8							

Table S3. Protocol of mild-intensity ET
