# Supplementary material for: Capsiate Intake with Exercise Training Additively Reduces Fat Deposition in Mice on a High-Fat Diet, but Not without Exercise Training
Source: Int J Mol Sci. 2021 Jan 14;22(2):769. doi: 10.3390/ijms22020769 (PMC7828664; doi:10.3390/ijms22020769)
Supplement: Supplementary file 1 [file ijms-22-00769-s001.zip › Supplementary material S1~S4/S4.pdf]

Primary antibody				Secondary antibody		
Target	Molecular Weight (kDa)	Concentration	Cat. No	Target	Concentration	Cat. No
UCP1	32	1:1000	sc-6529	anti-goat	1:2000	sc-2354
MDH2	35	1:25000	ab96193	anti-rabbit	1:50000	sc-2357
CS	52	1:25000	ab96600		1:50000	
PGC1α	91	1:2000	ab54481		1:4000	
GLUT4	55	1:3000	ab654		1:6000	
β2AR	46	1:2000	ab182136		1:20000	
β3AR	43	1:1000	ab94506		1:10000	
HXK2	102	1:4000	sc-130358	anti-mouse	1:8000	sc-516102
β-actin	42	1:1000	sc-47778		1:2000	
GAPDH	36	1:1000	sc-35062		1:2000	

Table S4. Information of used antibodies in the study.
